# Long-term safety outcomes and patient preferences for home-based intravenous enzyme replacement therapy (ERT) in Pompe disease and Mucopolysaccharidosis Type I (MPS-I): final results of two-year observation

**DOI:** 10.1186/s13023-025-04108-1

**Published:** 2025-11-21

**Authors:** Antonio Toscano, Olimpia Musumeci, Michele Sacchini, Sabrina Ravaglia, Gabriele Siciliano, Agata Fiumara, Elena Verrecchia, Rita Fischetto, Grazia Crescimanno, Roberta Taurisano, Annalisa Sechi, Serena Gasperini, Vittoria Cianci, Lorenzo Maggi, Filippo Brighina, Rita Barone, Annalia Cianflone, Marta Balzarini, Rossella Parini, Maurizio Scarpa

**Affiliations:** 1 Department of Clinical and Experimental Medicine, University of Messina, AOU Policlinico “G. Martino”, ERN-NMD Center for Neuromuscular Disorders of Messina, Messina, Italy; 2 Unit of Neurology and Neuromuscular Disorders, Department of Clinical and Experimental Medicine, University of Messina, Messina, Italy; 3 Metabolic Diseases and Neuromuscular Unit, Meyer Children’s Hospital IRCCS, Firenze, Italy; 4 IRCCS Fondazione Istituto Neurologico Nazionale C. Mondino, Pavia, Italy; 5 Department of Clinical and Experimental Medicine, S. Chiara Hospital - University of Pisa, Pisa, Italy; 6 Department of Clinical and Experimental Medicine, University of Catania, Catania, Italy; 7 Department of Geriatrics, Orthopedics and Rheumatology, Fondazione Policlinico Universitario A. Gemelli IRCSS, Rome, Italy; 8 A.O.U. Policlinico Consorziale-PO Giovanni XXIII, UOC Malattie Metaboliche Genetiche, Bari, Italy; 9 Institute for Biomedical Research and Innovation (IRIB), National Research Council (CNR), Palermo, Italy; 10 Metabolic Disease and Hepatology Division, Bambino Gesù Children’s Hospital, Rome, Italy; 11 Regional Coordinating Center for Rare Diseases, University Hospital of Udine, Udine, Italy; 12 Pediatrics, Fondazione IRCCS San Gerardo dei Tintori, Monza, Italy; 13 Great Metropolitan Hospital “Bianchi Melacrino Morelli” – Neurology, Reggio Calabria, Italy; 14 Neuroimmunology and Neuromuscular Diseases Unit, Fondazione IRCCS Istituto Neurologico Carlo Besta, Milano, Italy; 15 AOU Policlinico P. Giaccone di Palermo, Palermo, Italy; 16 Child Neuropsychiatry Unit, Department of Clinical and Experimental Medicine, University of Catania, Catania, Italy; 17 IRCCS San Gerardo dei Tintori Foundation, - Rare Disease Unit, Monza, Italy; 18 Pediatric Department, ARNAS G. Brotzu, Cagliari, Italy

**Keywords:** Enzyme replacement therapy, Pompe disease, Home therapy, Mucopolysaccharidosis, Treatment adherence, Safety

## Abstract

**Background:**

Pompe disease and Mucopolysaccharidoses Type I (MPS-I) are lysosomal disorders caused by a deficiency of α-glucosidase and alpha-L-iduronidase, respectively. The mainstay of treatment is enzyme replacement therapy (ERT), a life-long treatment that requires regular I.V. infusions. Hospital-based therapy can, however, negatively impact quality of life over time. The purpose of the HomERT study (Home infusions of ERT) was to evaluate the safety, treatment compliance, and treatment satisfaction related to home therapy of Pompe disease patients with Myozyme^®^ (alglucosidase alfa) and MPS-I patients with Aldurazyme^®^ (laronidase). The final results are presented in this paper.

**Results:**

The HomERT study was a multicenter, non-interventional, minimum 12-month prospective observational, double-cohort study that analyzed 56 patients from 14 sites in Italy from October 2021 to February 2024: cohort A (Pompe disease − 47 patients) and cohort B (MPS-I − 9 patients: 6 Hurler/Scheie, 3 Scheie). During the observation period, the mean (SD) number of missed infusions was 5.8 (3.92) in cohort A and 3.0 (3.52) in cohort B, corresponding to a mean (SD) of missed infusions of 19.8 (32.7)% and 4.1 (4.2)%, respectively, versus the number of planned infusions. Only 2 patients in cohort A returned to the hospital setting due to “adverse event” and “other” reasons. A total of 13 Adverse Drug Reactions (ADR) were reported during the home-care setting before and after enrollment. The average number of ADRs per patient was 0.2 (1.46) in cohort A and 0.2 (0.67) in group B, and the rate of ADRs/year was 0.15 (95% CI: 0.06; 0.34) in cohort A and 0.06 (95% CI: 0.01; 0.38) in cohort B. The majority of patients preferred home-based infusions (cohort A: 93.6%; cohort B: 88.9%), and the main reason was attributed to treatment convenience (cohort A: 93.6%; cohort B: 100%). Despite the underlying conditions, most patients self-evaluated their health as “Fair” in cohort A (36.2%) and “Good” in cohort B (44.5%).

**Conclusion:**

The use of ERT with α-glucosidase and alpha-L-iduronidase alfa remains a strong candidate for home therapy, with favorable safety profile, improved treatment ERT compliance, and patient satisfaction. NCT05073783.

**Supplementary Information:**

The online version contains supplementary material available at 10.1186/s13023-025-04108-1.

## Introduction

Lysosomal diseases (LDs) are a group of inherited, rare, progressive metabolic disorders characterized by deficiencies of lysosomal enzymes, resulting in the accumulation and abnormal processing of various substrates in tissues and organs [1–3]. Pompe disease (Glycogen Storage Disease type II, α-glucosidase deficiency), and Mucopolysaccharidosis Type I (MPS-I) (Hurler, Hurler/Scheie or Scheie Disease) are caused by a deficiency of α-glucosidase and alpha-L-iduronidase, respectively [2]. Due to these deficiencies, Pompe disease and MPS-I are multisystem disorders with storage of glycogen mainly in skeletal and cardiac muscle in the first and storage of glycosaminoglycans (GAGs) in virtually all tissues and organs in the second [2–4]. Pompe disease may be distinguished in infantile (IOPD) and late onset (LOPD) Pompe disease, and MPS-I is generally subdivided in three subgroups: Hurler, Hurler/Scheie and Scheie according to the severity of the disease (Scheie is the less severe form).

For both disorders, enzyme replacement therapy (ERT) with α-glucosidase (Myozyme^®^) for Pompe disease and laronidase (Aldurazyme^®^) for MPS-I [2, 5, 6] are available. Therapeutic options were limited to symptoms management and, only for the severe form of MPS-I, hematopoietic stem cell transplantation (HSCT), until ERT became available as etiologic treatment. ERT does not readily penetrate into the central nervous system (CNS) but partially prevents the irreversible somatic organ damage associated with progression [7, 8]. It was found that patients without treatment or with poor treatment compliance showed a more rapid progression of the disease, and the prolonged interruptions of ERT could cause a loss of clinical benefits as well as a significant worsening of clinical status [7–12]. Therefore, it is critical to maintain ERT to prevent progression of disease in the absence of adverse events.

ERT, a life-long treatment, requires regular infusion and is generally administered under the supervision of trained healthcare professionals due to safety concerns. Hospital-based therapy can however negatively impact the quality of life over time, while it was reported that home-based ERT might offer greater convenience and make patients and their families feel less stressed [13–15].

The advent of the Coronavirus Disease-19 (COVID-19) pandemic had a negative impact on the continued care and treatment of patients with LDs and caused the need of a switch in treatment patterns [16–18]. Furthermore, previous studies that investigated the impact of COVID-19 on treatment patterns in patients with LDs reported that the majority of patients received their infusions without interruptions under the home-setting and tended to prefer home-based therapy, even after the pandemic, in terms of quality of care [17, 18].

We report the final results from the HomERT study, which evaluated long-term safety, treatment compliance, and treatment satisfaction related to home therapy of Pompe disease patients with Myozyme^®^ (alglucosidase alfa) and MPS-I patients with Aldurazyme^®^ (laronidase) in a real-world setting in Italy.

## Methods

### Study design

The HomERT study was an Italian, multicenter, non-interventional, double-cohort study with both retrospective and prospective data collection to obtain information related to the safety and patient satisfaction of treating Pompe disease and MPS-I with ERT in a home-care setting. Data on patient reports, infusion characteristics, and adverse events (AEs) from Italian patients with Pompe disease and MPS-I who started ERT infusions at home were collected from October 2021 to February 2024. The final analysis included double cohort data with the retrospective data collected before October 2021 (enrollment) and the prospective data collected during at least 12 months of observation after enrollment.

During the visits conducted every 6–12 months, Investigators collected information on patient-reported outcomes and recorded any documented clinical data during the home infusions. The patient-reported outcomes (PRO) were conducted using a questionnaire to assess patient satisfaction related to the home infusions. The interim results of the study have previously been published [19]. The dosage and dosage regimen were in accordance with the Summary of Product Characteristics (SmPC).

### Study population

Eligible participants who had provided written informed consent were enrolled in the study. Home-based ERT was initiated following discussion and joint agreement between patient and physician, and was authorized by Italian regulations (including Agenzia Italiana del Farmaco (AIFA) authorization 341/2020 for Pompe disease patients and MPS-I patients). According to AIFA authorization, ERT could be administered in a home-care setting after at least 6–12 months of ERT in the hospital if ERT was proven safe and no recent infusion-associated reactions (IAR) had occurred. Significant chronic respiratory disease (e.g., % forced vital capacity ≤ 4O%) had to be stable and the patient in good overall clinical condition. The home-infusion team had to include a specialized physician available by telephone and one or two nurses trained in the pathology, ERT and possible adverse reactions.

The enrolled population included 56 patients from 14 sites in Italy and was categorized into 2 cohorts (cohort A and cohort B). Cohort A included Pompe disease patients with confirmed acid alpha-glucosidase (GAA) enzyme deficiency receiving Myozyme^®^ (alglucosidase alfa), and cohort B included MPS-I Hurler/Scheie and Scheie patients with confirmed deficiency of alpha-L-iduronidase receiving Aldurazyme^®^ (laronidase) in a home-care infusion setting according to AIFA-authorized clinical practice, Summary of Product Characteristics, and the approved risk management plan document. The patients in both cohorts were already in the home-based infusion setting prior to enrollment or selected to transfer to the home-based infusion setting at enrollment.

### Statistical methods

Descriptive statistics (n, mean, median, standard deviation (SD), range, min, max for continuous variables, and count and percentage for categorical variables) were used to summarize treatment exposure, safety outcomes, and patient characteristics. Data were analyzed and presented by cohort (cohort A and cohort B). Data from all sites were pooled and were analyzed and presented by cohort.

The primary analysis evaluated the incidence of treatment-emergent adverse events (TEAEs), including IARs, based on seriousness and relationship to the treatment. All TEAEs were summarized by system organ class (SOC) and preferred term (PT) according to the Medical Dictionary for Regulatory Activities (MedDRA) and maximum severity. In addition, extent of usage of concomitant medication and change in the use of concomitant medication in case of non-tolerated infusion were considered. The secondary analysis evaluated patient satisfaction by means of a questionnaire, and treatment compliance was assessed based on the number of missed infusions vs. planned and/or return to the hospital setting (with reasons). Analyses were performed on demographics and medical history according to MedDRA. Results of vital signs and laboratory parameters were categorized as low/normal/high based on clinical normal ranges, and abnormal values were flagged. Abnormalities on physical examination were also flagged. Statistical analyses were performed by means of SAS^®^ release 9.4 (SAS Institute, Inc., Cary, NC, USA).

## Results

### Participants’ disposition, socio-demographic and clinical characteristics

A total of 56 patients were enrolled in 2 cohorts. Of these, 47 patients made up cohort A (Pompe disease) and 9 patients cohort B (MPS-I). An overview of demographics and baseline characteristics is illustrated in Table 1.

**Table 1:** Baseline demographics and disease characteristics (Enrolled population)

	Cohort A (*N* = 47)	Cohort B (*N* = 9)	Total (*N* = 56)
**Demographics**			
Age (years)			
Mean (SD)	46.1 (23.99)	23.1 (11.03)	42.4 (23.91)
Q1; Q3	24; 65	15; 27	20; 64
Range	3; 85	9; 43	3; 85
Age, n (%)			
0–18 years	9 (19.15)	3 (33.33)	12 (21.43)
19–35 years	8 (17.02)	4 (44.44)	12 (21.43)
36–55 years	9 (19.15)	2 (22.22)	11 (19.64)
56–70 years	15 (31.91)	0	15 (26.79)
over 70 years	6 (12.77)	0	6 (10.71)
Sex, n (%)			
Female	23 (48.94)	3 (33.33)	26 (46.43)
Male	24 (51.06)	6 (66.67)	30 (53.57)
**Disease Characteristics**			
Diagnosis, n (%)			
MPS-I disease			
Hurler – Scheie	0	6 (66.67)	6 (10.71)
Scheie for MPS-I	0	3 (33.33)	3 (5.36)
Pompe disease			
Infantile onset Pompe disease	8 (17.02)	0	8 (14.29)
Late onset Pompe disease	39 (82.98)	0	39 (69.64)
Cognitive delay, n (%)			
No	47 (100.00)	7 (77.78)	54 (96.43)
Yes	0	2 (22.22)	2 (3.57)
Age at diagnosis (years)			
Mean (SD)	31.4 (23.45)	8.0 (7.86)	27.6 (23.33)
Q1; Q3	4.3; 52.2	4.1; 7.0	4.2; 49.5
Range	0; 67	2; 28	0; 67
Age at start of ERT administration in hospital setting (years)			
Mean (SD)	35.6 (24.16)	10.7 (8.26)	31.6 (24.14)
Q1; Q3	9.1; 57.1	5.5; 10.3	7.2; 53.9
Range	0; 73	4; 29	0; 73
Time from diagnosis to start of ERT administration in hospital setting (months)			
Mean (SD)	52.6 (64.95)	34.2 (48.37)	49.6 (62.57)
Q1; Q3	1.8; 101.9	6.1; 42.0	4.1; 88.7
Range	0; 211	4; 154	0; 211
**Patients starting ERT administration in a home-care setting**, n (%) *	**46 (97.87)**	**9 (100.00)**	**55 (98.21)**
Reasons for switching to home-care setting, n (%) *			
Center distant	4 (8.70)	0	4 (7.27)
COVID-19 related	24 (52.17)	2 (22.22)	26 (47.27)
Other	1 (2.17)	2 (22.22)	3 (5.45)
Patient request	17 (36.96)	5 (55.56)	22 (40.00)
Age at start of ERT administration in a home-care setting (years)*			
Mean (SD)	45.8 (23.65)	18.5 (12.68)	41.4 (24.37)
Q1; Q3	22.3; 64.6	7.6; 25.5	17.5; 62.7
Range	2; 83	5; 41	2; 83
Time from diagnosis to start of ERT administration in home-care setting (months)*			
Mean (SD)	167.7 (96.91)	127.3 (115.63)	161.1 (100.17)
Q1; Q3	83.2; 247.5	17.6; 169.5	82.5; 247.5
Range	11; 358	15; 326	11; 358
Time to switch to home-care setting (months)*			
Mean (SD)	114.0 (51.60)	93.1 (78.85)	110.6 (56.57)
Q1; Q3	80.0; 158.6	12.4; 146.9	65.1; 158.6
Range	9; 200	8; 208	8; 208
Time from start of ERT administration in homecare setting to screening (months)*			
Mean (SD)	16.1 (9.20)	62.5 (34.77)	23.7 (23.45)
Q1; Q3	8.1; 23.4	28.3; 92.6	9.0; 27.2
Range	0; 32	26; 116	0; 116
Patients who started the ERT administration in homecare setting prior to enrollment, n (%)*			
No	1 (2.13)	0	1 (1.79)
Yes	46 (97.87)	9 (100.00)	55 (98.21)
**Other Medical Conditions**			
Patients with prior medical conditions, n (%)	24 (51.06)	8 (88.89)	32 (57.14)
Patients with ongoing medical conditions, n (%)	40 (85.11)	7 (77.78)	47 (83.93)
Patients reporting the presence of any significant respiratory disease, n (%)	25 (53.19)	2 (22.22)	27 (48.21)
Patients with evidence of serious obstructive airway disease, n (%) ^$^			
Other	9 (36.00)	2 (100.00)	11 (40.74)
Respiratory Failure	16 (64.00)	0	16 (59.26)
Predicted forced vital capacity, (%) ^$^			
n	24	1	25
Mean (SD)	49.8 (18.46)	38.0	49.3 (18.22)
Q1; Q3	35.0; 66.0	38.0; 38.0	36.0; 64.0
Range	17; 82	38; 38	17; 82

*Q1 = 1st quartile; Q3 = 3rd quartile; SD = Standard Deviation.*

*Percentages were computed on patients belonging to the Enrolled population within each considered group.*

* Computed only for patients starting the homecare setting administration. One patient started ERT administration in homecare setting at the screening visit.

^*$*^ *Computed only for patients reporting the presence of any significant respiratory disease.*

*Cohort A is consisting of Pompe disease patients receiving Myozyme in a home-care setting*,* while cohort B is composed of MPS-I patients receiving Aldurazyme in a home-care setting.*

***Age at start date of ERT administration in hospital setting (years)*** *is calculated as: age at diagnosis + (start date of ERT in hospital setting – date of diagnosis)/365.25.*

***Time from diagnosis to start of ERT administration in hospital setting (months)*** *is calculated as: (start date of ERT administration in hospital setting - date of diagnosis)/30.4375.*

***Age at start of ERT administration in a home-care setting (years)*** *is calculated as: age at diagnosis + (start date of ERT in home-care setting – date of diagnosis)/365.25.*

***Time from diagnosis to start of ERT administration in home-care setting (months)*** *is calculated as: (start date of ERT administration in home-care setting - date of diagnosis)/30.4375.*

***Time to switch to home-care setting (months)*** *is calculated as: (start date of ERT administration in home-care setting – start date of ERT administration in hospital setting)/30.4375.*

***Time from start of ERT administration in home-care setting to screening (months)*** *is calculated as: (Screening visit - start date of ERT administration in homecare setting)/30.4375.*

The mean (SD) age was 46.1 (24) years in cohort A and 23.1 (11) years in cohort B, with the majority aged 56–70 years in cohort A (15 patients, 31.9%), and 19–35 years in cohort B (4 patients, 44.4%). The proportion of male patients was higher in both cohorts (51.1% and 66.7%, respectively). In cohort A, 83% of patients had LOPD and 17% had IOPD, with no cognitive delay. In cohort B, 66.7% of patients were reported as Hurler – Scheie and 33.3% as Scheie, with 22.2% with cognitive delay (all Hurler/Scheie). The mean (SD) age at diagnosis was 31.4 (23.5) years in cohort A and 8 (7.9) years in cohort B. The mean (SD) age at start of ERT administration in a hospital setting was 35.6 (24.2) years in cohort A and 10.7 (8.3) years in cohort B; and mean (SD) time from diagnosis to start of ERT administration in a hospital setting was 52.6 (65.0) months in cohort A and 34.2 (48.4) months in cohort B.

All the patients belonging to cohort B and all but one of cohort A started ERT at the hospital initially and were switched to home therapy. The most frequent reasons for switching to home therapy were COVID-related (52.2%) and patient request (37.0%) in cohort A, and patient request (55.6%), other reasons (medical decision and improved patient status), and COVID-related (22.2%, respectively) in cohort B. The mean (SD) time from diagnosis to start of ERT administration in a home setting was 167.7 (96.9) months in cohort A and 127.3 (115.6) months in cohort B.

Twenty-five patients (53.2%) in cohort A and 2 patients (22.2%) in cohort B reported significant respiratory disease. In cohort A, 22 (56.4%) were LOPD patients, and 3 (37.5%) were IOPD patients (data not shown). Sixteen (64.00%) of the 25 patients in cohort A with significant respiratory disease and none in cohort B experienced respiratory failure, while the others reported other signs of serious obstructive airway disease.

### Preferences for home therapy based on safety outcomes and tolerability

A summary of home infusions and ADRs occurring prior to enrollment is shown in Table 2. Three patients (6.5%) in cohort A and 2 patients (22.2%) in cohort B reported at least one prior ADR. Among the 7 ADRs reported for both cohorts (4 and 3, respectively), 3 ADRs in cohort A and 1 ADR in cohort B occurred in a hospital setting: non-serious mild “erythema”, serious moderate “urticaria”, and serious moderate “dyspnea” in cohort A (3 LOPD patients) and non-serious mild “rash” in cohort B (1 patient with Hurler/Scheie). The mean (SD) number of ADRs observed per patient in the hospital setting was 0.06 (0.32) (range: 0;2) in cohort A and 0.11 (0.33) (range: 0; 1) in cohort B, with a rate of ADRs/year of 0.006 (95% CI: 0.002; 0.020) in cohort A and 0.014 (95% CI: 0.002; 0.102) in cohort B. In the home setting, one ADR of non-serious mild “urticaria” was reported in 1 patient in cohort A, and 2 ADRs of non-serious mild “pyrexia” were reported in 1 patient in cohort B.

**Table 2:** Summary of prior home infusion and ADRs; enrolled patients who started treatment in a home setting before enrollment

	Cohort A (*N* = 47)	Cohort B (*N* = 9)	Total (*N* = 56)
**Number of patients administered prior home infusion**	46	9	55
**Number of dilutions performed by patients**			
Mean (SD)	1.0 (0.00)	1.0 (0.00)	1.0 (0.00)
Q1; Q3	1.0; 1.0	1.0; 1.0	1.0; 1.0
Range	1; 1	1; 1	1; 1
**Any change in dilution infusion**,** n (%)**			
Yes	0	0	0
No	46 (100.00)	9 (100.00)	55 (100.00)
**Prior home infusion by rate of administration**,** n (%)**			
Every 2 weeks	41 (89.13)	0	41 (74.55)
Other	1 (2.17)	0	1 (1.82)
Weekly	4 (8.70)	9 (100.00)	13 (23.64)
**Infusions by administration of any pre-medication**,** n (%)**			
No	36 (78.26)	6 (66.67)	42 (76.36)
Yes	10 (21.74)	3 (33.33)	13 (23.64)
**Number of missed ERT infusions during ERT administration** **in a home-care setting**			
Mean (SD)	0.6 (1.22)	4.6 (5.5)	1.3 (2.80)
Q1; Q3	0.0; 1.0	0.0; 6.0	0.0; 1.0
Range	0; 4	0; 16	0; 16
**Average duration of infusion (hours)** ^**a**^			
Mean (SD)	4.5 (1.09)	3.6 (0.53)	4.4 (1.08)
Q1; Q3	4.0; 5.0	3.0; 4.0	4.0; 5.0
Range	3; 7	3; 4	3; 7
**Prior ADRs n (%)** ^**b**^			
Number of prior Adverse Drug Reactions	3 (6.52)	2 (22.22)	5 (9.09)
Number of prior Adverse Drug Reactions by setting			
Home infusion	1 (25.00)	2 (66.67)	3 (42.86)
Hospital infusion	3 (75.00)	1 (33.33)	4 (57.14)
Serious prior Adverse Drug Reactions			
No	2 (50.00)	3 (100.00)	5 (71.43)
Yes	2 (50.00)	0	2 (28.57)
Severity of prior ADRs			
Mild	2 (50.00)	3 (100.00)	5 (71.43)
Moderate	2 (50.00)	0	2 (28.57)
* MedDRA System organ class/*			
Preferred term ^c^			
* General disorders and administration site conditions*	0	1 (11.11)	1 (1.82)
Pyrexia	0	1 (11.11)	1 (1.82)
* Immune system disorders*	1 (2.17)	0	1 (1.82)
Urticaria	1 (2.17)	0	1 (1.82)
* Respiratory*,* thoracic*,* and mediastinal disorders*	1 (2.17)	0	1 (1.82)
Dyspnea	1 (2.17)	0	1 (1.82)
* Skin and subcutaneous tissue disorders*	2 (4.35)	1 (11.11)	3 (5.45)
Erythema	1 (2.17)	0	1 (1.82)
Rash	0	1 (11.11)	1 (1.82)
Urticaria	1 (2.17)	0	1 (1.82)

*ERT = Enzyme Replacement Therapy; ADR = Adverse Drug Reaction*,* MedDRA = Medical Dictionary for Regulatory Activities; Q1 = 1st quartile; Q3 = 3rd quartile; SD = Standard Deviation*

*Percentages were computed on patients belonging to the enrolled population who started the home-care setting before enrollment within each considered group; Cohort A consists of Pompe disease patients receiving Myozyme in a home-care setting*,* while cohort B is composed of MPS-I patients receiving Aldurazyme in a home-care setting.*

^*a*^ *Duration of infusion was the time spent performing home infusions (hours) reported by patients for each performed dilution.*

^*b*^ *Computed on the total number of prior ADRs occurred within each considered group; Each subject could have more than one prior Adverse Drug Reaction*,* but they are counted only once for each condition/row.*

^*c*^ *Terms were coded using MedDRA*,* version 24.0.*

TEAEs observed after initiation of ERT in a home setting are summarized in Table 3. A total of 45 TEAEs were reported by 21 patients in the enrolled population: 39 TEAEs were reported by 17 patients in cohort A and 6 TEAEs by 4 patients in cohort B.

**Table 3:** Summary of TEAEs (Enrolled Population)

	Cohort A (*N* = 47)	Cohort B (*N* = 9)	Total (*N* = 56)
**Number of patients with at least one of the following, *n*** **(%), E**			
TEAEs	17 (36.17), 39	4 (44.44), 6	21 (37.50), 45
Pompe disease (cohort A)			
Infantile onset (*n* = 8)	5 (62.50), 11	-	17 (36.17), 39
Late onset (*N* = 39)	12 (30.77), 28	-	
MPS-I (cohort B)			
Hurler – Scheie (*N* = 6)	-	3 (50.00), 5	4 (44.44), 6
Scheie for MPS-I (*N* = 3)	-	1 (33.33), 1	
Treatment-related TEAEs	1 (2.13), 10	0, 0	1 (1.79), 10
Serious TEAEs	6 (12.77), 9	1 (11.11), 1	7 (12.50), 10
Treatment-related serious TEAEs	0, 0	0, 0	0, 0
Severe TEAEs	3 (6.38), 3	1 (11.11), 1	4 (7.14), 4
Treatment-related severe TEAEs	0, 0	0, 0	0, 0
TEAEs leading to withdrawal	1 (2.13), 2	0, 0	1 (1.79), 2
TEAEs of special interest	1 (2.13), 10	0, 0	1 (1.79), 10
Treatment emergent infusion reaction associated with study drug	1 (2.13), 10	0, 0	1 (1.79), 10
**Treatment-related TEAEs n (%); enrolled patients**^**a**^, **n (%)**,** E**			
Number of patients with Treatment-related TEAEs	1	0	0
*MedDRA System organ class/*			
Preferred term ^b^			
*Injury*,* poisoning and procedural complications*	1 (2.13), 10	0	1 (1.79), 10
Infusion related reaction	1 (2.13), 10	0	1 (1.79). 10
**ADR incidence**			
Number of total Adverse Drug Reactions	11	2	13
Adverse Drug Reactions per patient			
Mean (SD)	0.2 (1.46)	0.2 (0.67)	0.2 (1.36)
Q1; Q3	0; 0	0; 0	0; 0
Range	0; 10	0; 2	0; 10
Rate of ADRs/years [95% CI]^c^	0.15 [0.06; 0.34]	0.06 [0.01; 0.38]	0.13 [0.06; 0.29]

*ADRs = Adverse Drug Reactions; MPS-I = Mucopolysaccharidoses Type I; TEAE = Treatment Emergent Adverse Event.*

*Percentages were computed on patients belonging to the Enrolled population within each group considered.*

*Cohort A consists of Pompe disease patients receiving Myozyme in a home-care setting*,* while Cohort B is composed of MPS-I patients receiving Aldurazyme in a home-care setting.*

*Each patient could experience more than one TEAEs and were counted only once in the system organ class or preferred term category.*

^*a*^ *ADRs collected during the retrospective period (i.e.*,* ADRs that occurred during the last home-care infusion before enrolment in the study) and ADRs collected during the prospective period (i.e.*,* ADRs after study enrolment) were considered.*

^*c*^ *Terms were coded using MedDRA*,* version 24.0.*

^c^ *Rates and relative 95% CIs have been estimated using a Poisson regression model or*,* in case of overdispersion*,* a Negative Binomial regression model.*

Breaking down by type of disease for each cohort, 5 (62.5%) out of 8 IOPD patients and 12 (30.8%) out of 39 LOPD patients experienced at least one TEAE in cohort A; 3 (50%) of 6 patients with MPS-I Hurler/Scheie syndrome and 1 (33.3%) of 3 patients with MPS-I Scheie experienced at least one TEAE in cohort B. Only one patient (1.8%) (LOPD) experienced treatment-related TEAEs or AEs of special interest: the patient experienced 10 “infusion-related reactions”, but none were reported as severe or serious events.

A total of 13 ADRs (3 before and 10 after enrollment) were reported for 3 patients while in a home-care setting. The mean (SD) number of ADRs observed per patient was 0.2 (1.46) (range: 0;10) in cohort A and 0.2 (0.67) (range: 0; 2) in group B, with the rate of ADRs/year in a home-care setting of 0.15 (95% CI: 0.06; 0.34) in cohort A and 0.06 (95% CI: 0.01; 0.38) in cohort B.

Two deaths (cerebral hemorrhage and “death”) not related to treatment occurred during the study. Among the 10 serious TEAEs, 9 events were reported in 6 patients (12.8%) in cohort A, including “death”, “pneumonia”, “staphylococcal infection”, “femur fracture”, “head injury”, “respiratory failure”, “cerebral hemorrhage”, “transient ischemic attack”, and “presyncope”. One event of “cardiac arrest” was reported in 1 patient (11.1%) in cohort B but was not related to the treatment. (Supplementary Table 1). The severity of most TEAEs was mild or moderate except for 4 severe TEAEs: 3 were reported in cohort A (“death”, “femur fracture”, “cerebral hemorrhage”) and one (“cardiac arrest”) in cohort B, but none were treatment related (Supplementary Table 2).

Thirty-eight patients (80.9%) in cohort A and 7 patients (77.8%) in cohort B had at least one concomitant medication, and only one IOPD patient (2.1%) in cohort A received an immunosuppressant (sirolimus) due to intolerance to the drug infusion.

No significant trends or changes were observed in vital signs and laboratory parameters. Among the physical examination parameters, the notable changes from the screening visit to the end of the study were reported in the respiratory, abdomen, nervous, and musculoskeletal systems. A few patients showed a worsening of the respiratory, nervous, and musculoskeletal systems and others an improvement of abdominal signs/symptoms. No other relevant changes were reported (Supplementary Table 3).

### ERT compliance and treatment satisfaction

#### ERT compliance

Before enrollment in this study, 41 patients (89.1%) in cohort A received ERT every 2 weeks, and all 9 patients (100%) in cohort B received ERT weekly in a home-care setting. The mean (SD) number of planned total infusions in a home-care setting based on treatment regimen prior to enrollment was 37.1 (24.1) infusions for cohort A and 271.8 (151.2) infusions for cohort B. The mean (SD) number of missed ERT infusions in a home-care setting before enrollment was 0.6 (1.2) infusions in cohort A and 4.5 (1.1) infusions in cohort B. The mean infusion duration was 4.5 (1.1) hours in cohort A and of 4.5 (1.09) hours in cohort B (Table 2).

ERT compliance for patients still in a home-care setting after enrollment is shown in Table 4. Among the 56 enrolled patients, 54 (98.21%) were included in the analysis, whereas 2 patients in cohort A performed only the screening visit before death or were lost to follow-up. The mean (SD) number of planned total infusions after enrollment was 45.7 (31.1) infusions for cohort A and 64.4 (13.1) infusions for cohort B. Eight patients (17.8%) in cohort A and 6 patients (66.7%) in cohort B missed at least one infusion during the study. The mean (SD) number of missed infusions was 5.8 (3.9) in cohort A and 3.0 (3.5) in cohort B, corresponding to a mean (SD) proportion of missed infusions of 19.8 (32.7)% and 4.1 (4.2)%, respectively. The reasons for missed infusions were: “Other” (5 patients, 62.50%), “Patient/Parent Decision” (4 patients, 50.00%), and “Adverse Event” (3 patients, 37.50%) in cohort A; “Other” (5 patients, 83.33%) and “Patient/Parent Decision” (3 patients, 50.00%) in cohort B. Only 2 patients in cohort A returned to a hospital setting after enrollment due to AE (1 patient) and another reason (1 patient).

**Table 4:** Treatment compliance and exposure of Home-based ERT during the study (Enrolled Population)

	Cohort A (*N* = 47)	Cohort B (*N* = 9)	Total (*N* = 56)
**Treatment Compliance of Home-based ERT**			
Patients providing compliance information, n (%)	45 (95.74)	9 (100.00)	54 (96.43)
Number of total planned infusions			
Mean (SD)	45.7 (31.07)	64.4 (13.12)	48.8 (29.61)
Q1; Q3	28; 49	55; 79	28; 56
Range	3; 172	51; 83	3; 172
Any missed infusions?, n (%)			
No	37 (82.22)	3 (33.33)	40 (74.07)
Yes	8 (17.78)	6 (66.67)	14 (25.93)
Number of total missed infusions			
n	8	6	14
Mean (SD)	5.8 (3.92)	3.0 (3.52)	4.6 (3.88)
Q1; Q3	2.5; 9.0	1.0; 3.0	2.0; 7.0
Range	2; 12	1; 10	1; 12
Proportion of total missed infusions (%)			
n	8	6	14
Mean (SD)	19.8 (32.70)	4.1 (4.20)	13.1 (25.44)
Q1; Q3	5.75; 14.38	1.52; 3.80	3.64; 11.29
Range	3.77; 100.00	1.21; 12.35	1.21; 100.00
Reason of missed infusions, n (%)^a^			
Adverse Event	3 (37.50)	0	3 (21.43)
Patient / Parent Decision	4 (50.00)	3 (50.00)	7 (50.00)
Other	5 (62.50)	5 (83.33)	10 (71.43)
Return to hospital setting, n (%)			
No	43 (95.56)	9 (100.00)	52 (96.30)
Yes	2 (4.44)	0	2 (3.70)
Reason of return to hospital setting, n (%)^b^			
Adverse Event	1 (50.00)	0	1 (50.00)
Other	1 (50.00)	0	1 (50.00)
**Exposure to Home-based ERT** ^**c**^			
Total exposure to ERT treatment (months)			
Mean (SD)	146.1 (54.21)	171.7 (59.35)	150.2 (55.32)
Median	144.9	187.6	151.3
Q1; Q3	102.97; 195.81	137.23; 199.00	112.44; 198.83
Range	27.73; 239.01	53.62; 248.02	27.73; 248.02
Exposure to ERT treatment in home-care setting (months)			
Mean (SD)	32.3 (9.77)	78.6 (37.87)	39.7 (24.16)
Median	34.8	71.5	37.1
Q1; Q3	19.88; 40.58	45.14; 111.64	28.24; 41.28
Range	14.00; 46.19	40.44; 140.49	14.00; 140.49
Exposure to ERT treatment in home-care setting during the study (months)			
n	45	9	54
Mean (SD)	16.4 (6.41)	16.0 (4.31)	16.3 (6.08)
Median	15.5	13.3	15.3
Q1; Q3	12.26; 20.50	12.85; 19.02	12.26; 20.30
Range	0.03; 35.78	12.19; 24.15	0.03; 35.78

*ERT = Enzyme Replacement Therapy; Q1 = 1st quartile; Q3 = 3rd quartile; SD = Standard Deviation.*

*Percentages were computed on patients who provided compliance information after the screening visit and belonged to the Enrolled population within each group.*

*Cohort A consists of Pompe disease patients receiving Myozyme in a home-care setting*,* while Cohort B is composed of MPS-I patients receiving Aldurazyme in a home-care setting.*

^*a*^ *Percentages were computed on patients belonging to the Enrolled population who missed infusions within each group and each visit. Each patient could report more than one reason. Multiple occurrences of the same reason were counted only once.*

^*b*^ *Percentages were computed on patients belonging to the Enrolled population who returned to the hospital within each group and each visit. Each patient could report more than one reason. Multiple occurrences of the same reason were counted only once.*

*Note: Two patients performed only screening visit and discontinued the study before performing end of study visit (due to death and lost to follow-up)*,* therefore they did not provide any information about compliance.*

^*c*^ *Exposure to ERT treatment in home-care setting and exposure to ERT treatment in home-care setting during the study were not computed for 2 patients since no treatment start date in home-care setting after enrolment was available.*

***Total ERT exposure*** *= (study completion/discontinuation date – ERT start date + 1)/30.4375.*

***Exposure to ERT treatment in home-care setting was computed (in months)*** *: ERT exposure = (study completion/discontinuation date – ERT start date in home- care setting +1)/30.4375.

Exposure to ERT treatment in home-care setting during the study was computed*: *ERT exposure = (study completion/discontinuation date – screening date +1)/30.4375.*

* Exposure to ERT treatment in home-care setting and exposure to ERT treatment in home-care setting during the study were not computed for 2 patients since no treatment start date in home-care setting after enrolment was available.

#### Treatment satisfaction

Nearly all the patients (93.6%) in cohort A (IOPD: 100%, LOPD: 92.3%) and cohort B (88.9%) preferred to receive ERT infusions at home. Preference for home-care therapy was attributed to more treatment convenience (cohort A: 93.6%; cohort B: 100%), perception of less stressfulness (cohort A: 78.7%; cohort B: 66.7%), and fewer transportation requirements (cohort A: 68.1%; cohort B: 55.6%).

Despite the underlying condition (LD), a higher proportion of patients self-evaluated their health as “Fair” in cohort A (36.2%) and “Good” in cohort B (44.5%), and only 8 patients (17%) (all in cohort A) self-evaluated their health as “Poor” during the study. Of significance, 1 patient (2.1%) in cohort A and 2 patients (22.2%) in cohort B self-evaluated their health as “Excellent”, 7 patients (14.9%) in cohort A and 1 patient (11.1%) in cohort B as “Very good”, and 14 patients (29.8%) in cohort A and 4 patients (44.4%) in cohort B as “Good”. When comparing the improvement observed in physical health after receiving ERT at home to the hospital setting, the majority of patients in cohort A rated their physical health as “About the same” (22 patients, 46.8%) and “Much better now” (15 patients, 31.9%), while 8 patients (17%) rated it as “Somewhat better now”; patients in cohort B rated their physical health as “Much better now” (4 patients, 44.4%), “About the same” (3 patients, 33.3%), and “Somewhat better now” (2 patients, 22.2%) (Table 5).

**Table 5:** Impact of the home-based infusions on treatment satisfaction (Enrolled population)

	Cohort A (*N* = 47) *n* (%)	Cohort B (*N* = 9) *n* (%)	Total (*N* = 56) *n* (%)
Number of patients who completed the home infusion satisfaction questionnaire	47 (100.00)	9 (100.00)	56 (100.00)
Where do you prefer to take your ERT infusions?			
Hospital/clinic near your home	3 (6.38)	1 (11.11)	4 (7.14)
Home	44 (93.62)	8 (88.89)	52 (92.86)
Why do you prefer to take your ERT infusion in home setting? ^a^			
More convenient	44 (93.62)	9 (100.00)	53 (94.64)
Less stressful	37 (78.72)	6 (66.67)	43 (76.79)
Daily activities are less disrupted	20 (42.55)	5 (55.56)	25 (44.64)
Work/school are less disrupted	15 (31.91)	4 (44.44)	19 (33.93)
Family life is less disrupted	20 (42.55)	4 (44.44)	24 (42.86)
Less transportation needed (drive, take bus/taxi/train)	32 (68.09)	5 (55.56)	37 (66.07)
More clinical supervision	5 (10.64)	0	5 (8.93)
Feel less socially isolated	3 (6.38)	0	3 (5.36)
Other	0	1 (11.11)	1 (1.79)
In general, and despite your LD, would you say your health is:			
Excellent	1 (2.13)	2 (22.22)	3 (5.36)
Very good	7 (14.89)	1 (11.11)	8 (14.29)
Good	14 (29.79)	4 (44.44)	18 (32.14)
Fair	17 (36.17)	2 (22.22)	19 (33.93)
Poor	8 (17.02)	0	8 (14.29)
If you’re receiving ERT at home, how would you rate health in general compared to the period you received ERT in the hospital?			
Much better now	15 (31.91)	4 (44.44)	19 (33.93)
Somewhat better now	8 (17.02)	2 (22.22)	10 (17.86)
About the same	22 (46.81)	3 (33.33)	25 (44.64)
Missing	2 (4.26)	0	2 (3.57)
Time to last completed questionnaire (months)			
Mean (SD)	30.4 (10.78)	75.9 (36.10)	37.7 (23.88)
Q1; Q3	19.2; 40.0	45.1; 111.6	21.1; 41.0
Range	8.9; 45.0	40.4; 128.6	8.9; 128.6

*ERT = Enzyme Replacement Therapy*,* LD = Lysosomal Storage Disease.*

*Percentages were computed on patients belonging to the Enrolled population who completed the questionnaire within each group.*

*Cohort A consists of Pompe disease patients receiving Myozyme in a home-care setting*,* while Cohort B is composed of MPS-I patients receiving Aldurazyme in a home-care setting.*

^*a*^ *Percentages were computed on patients belonging to the Enrolled population within each group.*

* Each patient could select more than one option.

*Time to last completed questionnaire was computed (in months) as: (last questionnaire date - ERT start date in home- care setting + 1)/30.4375.*

*Of note: some patients completed only questionnaire at screening visit.*

## Discussion

These final results of the HomERT study program demonstrate that home-care therapy for Pompe disease and MPS-I Hurler/Scheie and Scheie can be an effective treatment strategy in Italy based on safety, ERT compliance, and treatment satisfaction.

ERT is the current standard treatment for Pompe disease and MPS-I, and aims to slow progression of disease [2, 20]. Therefore, it is critical to administer regular treatment because its interruption or poor therapeutic compliance leads to a deterioration of clinical parameters [21, 22]. The COVID-19 pandemic was a challenge for both health care and patients with LD, who missed their scheduled access to the clinics to get treatment [17, 21, 22]. The AIFA had temporarily and exceptionally authorized (341/2020) home infusion in Italy for patients with Pompe disease and MPS-I. However, the continuous risk and benefit balance of home-based therapy needed to be evaluated for permanent authorization (granted after completion of the study). Under the circumstances, some studies investigated the ERT pattern after the COVID-19 pandemic and demonstrated that most patients changed their treatment patterns from hospital-based to home-based therapy with higher satisfaction [17, 21, 22].

In the interim analysis of the HomERT study, more than 90% of patients were undergoing ERT in a home setting before enrollment. ERT was administered for an average of 14.6 months in cohort A and 69.5 months in cohort B [19]. Similarly, all patients included in the final analysis, except for 1 patient in cohort A, were treated with ERT in a home setting for an average of 16.1 months for cohort A and 62.5 months for cohort B.

Safety outcomes of home-setting ERT presented an acceptable tolerability and manageable safety profile. After enrollment, the patients were followed up an average of more than 12 months in both cohorts. In cohort A, the incidence of TEAEs was approximately two times higher in IOPD patients than in LOPD patients. However, given the absence of notable safety issues, including serious events, it cannot be concluded that there is clinical relevance in the different incidence of TEAEs between IOPD and LOPD patients. During this observation period, 10 ADRs related to treatment occurred in 1 LOPD patient in cohort A, and these events were all IARs. Thirteen ADRs were reported in the last home-care setting, including 10 ADRs after study enrollment and 3 ADRs before enrollment [19]. Focusing on seriousness and severity, the key factors for safety evaluation, the reported ADRs (“pyrexia” and “infusion-related reaction”) were mild, not serious, and did not require urgent medical intervention. All ADRs are consistent with those previously reported for the home-based infusion of ERT in patients with MPS-I [23] and Pompe disease [24]. Additionally, IARs, which may be a major concern in home-based ERT [25], occurred in only one Pompe disease patient (2.13%) and were not serious or severe. Of note, the incidence of IARs in Pompe disease patients treated with alglucosidase alfa was lower than that reported in other studies (~ 38%) [25–27].

The mean incidence of ADRs per patient was low (0.2), indicating that each patient experienced less than the average of 1 ADR. The incidence per year is under 1 ADR per year in both cohorts. The final data from this study suggest that alglucosidase alfa and laronidase can be administered as safely in home-care settings as in the hospital.

The patients were compliant with treatment at home. Given the mean of missed infusions, cohort A and cohort B patients were administered approximately 80% and 96% of the planned ERT, respectively. Only 3 patients did not receive the treatment due to the occurrence of AEs, and one patient in cohort A returned to hospital-based therapy due to a safety issue. Regardless of cohorts, approximately 98% of patients stated that home-based therapy increased their overall treatment satisfaction in terms of comfort and convenience while reducing their stress and transportation requirements. The results from this final analysis are consistent with the earlier published research on the at-home administration of ERT IV infusions, reporting that home-based therapy was more convenient and less stressful than hospital-based therapy [14].

Patients in both cohorts were optimistic about their health on ERT treatment administered in a home-care setting, which was used as a QoL indicator. Despite the LD manifestations, most patients conveyed satisfaction with their health condition compared to hospital-care therapy while receiving ERT under the home-setting infusion. Moreover, the available evidence on home-care ERT during the COVID-19 pandemic and outside of Italy also indicated that home-therapy reduces utilization of hospital resources and improves QoL [17, 27]. Likewise, this HomERT study demonstrated that home-based ERT can increase treatment compliance with comfortability and satisfaction.

An assessment of health resource utilization and Pharmacoeconomics did not fall into the scope of this study. However, home-based ERT may offer financial benefits for the healthcare system, including reduced use of hospital resources such as treatment rooms and nursing staff [28]. A budget impact assessment from the perspective of United States payers found that home ERT costs 25% to 50% less than ERT administered in outpatient infusion clinics or hospitals [29]. A study of the health care costs of home care ERT for patients with lysosomal storage disorders in Germany found that 98.5% of these costs were attributable to the infusion therapy and administration itself, and approximately 1.5% were attributable to personnel and travel costs, and therefore that moving ERT into the home environment is not expected to increase costs [30].

Health care professionals play a key role in home-based ERT. It is critical they offer support to both the patient and family and also provide a safe and comfortable environment throughout the process. Indeed, the nursing professional can build a relationship of trust, contributing to the acceptance and success of the procedure [31]. The home care nurse must meet the requirements for home care assistance specific for each lysosomal storage disorder and be trained in everything related to the ERT infusion, as well as how to handle anaphylactic reactions and other health emergencies.

One of the strengths of this study is that all recruited participants except for 1 were enrolled in Italy, minimizing ethnic and cultural bias that may confound patient preference. The advantage of using data obtained before and after enrollment was that it was possible to collect safety, treatment compliance, and satisfaction data for more than 12 months in a routine clinical setting under real-life conditions, which are more representative of the study population of interest and the clinical outcomes under observation. The main limitations of this study are attributed to its non-interventional and observational nature, which may involve patient selection bias, incomplete or missing data, lack of internal validity (no control group), difficulty in interpreting or verifying documented information, and variability between patients in the quality of documentation. Furthermore, treatment satisfaction assessed through patient responses is subjective and may involve a risk of recall bias. Hence, some outcomes might not be accurate. Almost all enrolled patients were already in a home infusion setting before enrollment, and the preference for home therapy over hospital therapy by an experienced person may differ from the judgment of a participant new to treatment. The lack of in-depth investigations encompassing a bigger cohort size or comparing the attitudes between young and adult patients could have added a new opinion to treatment preference.

## Conclusion

In summary, the majority of patients with Pompe disease and MPS-I contemplated home-based therapy to be more convenient, more flexible, and less stressful than hospital-based therapy. Our real-world data final analysis of the HomERT program proved that home-care ERT infusions of laronidase and alglucosidase alfa in patients with Pompe disease and MPS-I (mild or moderate forms) are associated with a favorable safety profile, marked by mild and infrequent treatment-related TEAEs, and consistent clinical findings. Additionally, we found high patient satisfaction and ERT compliance with receiving the infusions at home. As the newly approved ERT (Avalglucosidase alfa) has recently been outlined, home-care ERT infusions are likely to increase, also considering the recent EPOC recommendations [29]. Given the circumstances, ERT infusions of laronidase and alglucosidase alfa are still strong candidates for therapy with long-term results.

## Electronic supplementary material

Below is the link to the electronic supplementary material.

Supplementary Material 1

## Data Availability

The data that support the findings of this study are not openly available due to reasons of sensitivity and are available from the corresponding author upon reasonable request. Data are located in controlled access data storage at Sanofi s.r.l. Sole Shareholder.
