# Supplementary material for: Long-term safety outcomes and patient preferences for home-based intravenous enzyme replacement therapy (ERT) in Pompe disease and Mucopolysaccharidosis Type I (MPS-I): final results of two-year observation
Source: Orphanet J Rare Dis. 2025 Nov 21;20:639. doi: 10.1186/s13023-025-04108-1 (PMC12752199; doi:10.1186/s13023-025-04108-1)
Supplement: Supplementary file 1 — Supplementary Material 1 [file 13023_2025_4108_MOESM1_ESM.docx]

## Supplementary Tables and Figures

S. Table 1: Summary of Serious TEAE in Enrolled Population

|  | **Cohort A (N=47) n (%)** | **Cohort B (N=9) n (%)** | **Total (N=56) n (%)** |
| --- | --- | --- | --- |
| **Number of patients reporting serious TEAEs** | 6 (12.77) | 1 (11.11) | 7 (12.50) |
| *MedDRA System Organ Class* /  Preferred Term |  |  |  |
| *Cardiac disorders* | 0 | 1 (11.11) | 1 (1.79) |
| Cardiac arrest | 0 | 1 (11.11) | 1 (1.79) |
| *General disorders and administration site conditions* | 1 (2.13) | 0 | 1 (1.79) |
| Death | 1 (2.13) | 0 | 1 (1.79) |
| *Infections and infestations* | 1 (2.13) | 0 | 1 (1.79) |
| Pneumonia | 1 (2.13) | 0 | 1 (1.79) |
| Staphylococcal infection | 1 (2.13) | 0 | 1 (1.79) |
| *Injury, poisoning and procedural complications* | 2 (4.26) | 0 | 2 (3.57) |
| Femur fracture | 1 (2.13) | 0 | 1 (1.79) |
| Head injury | 1 (2.13) | 0 | 1 (1.79) |
| *Nervous system disorders* | 3 (6.38) | 0 | 3 (5.36) |
| Cerebral haemorrhage | 1 (2.13) | 0 | 1 (1.79) |
| Presyncope | 1 (2.13) | 0 | 1 (1.79) |
| Transient ischaemic attack | 1 (2.13) | 0 | 1 (1.79) |
| *Respiratory, thoracic and mediastinal disorders* | 1 (2.13) | 0 | 1 (1.79) |
| Respiratory failure | 1 (2.13) | 0 | 1 (1.79) |
| *TEAE=Treatment Emergent Adverse Event.   Percentages were computed on patients belonging to the Enrolled population within each considered group. Cohort A is consisting of Pompe disease patients receiving Myozyme in a home-care setting, while Cohort B is composed of MPS-I patients receiving Aldurazyme in a home-care setting. Patients with more than one TEAE or multiple occurrences of the same TEAE were counted only once in the System Organ Class or Preferred Term category.* | | | |

S. Table 2: Summary of TEAE by Maximum Severity in Enrolled Population

|  | **Cohort A (N=47)** | | | | **Cohort B (N=9)** | | | | **Total (N=56)** | | | |
| --- | --- | --- | --- | --- | --- | --- | --- | --- | --- | --- | --- | --- |
|  | **Mild n (%)** | **Moderate n (%)** | **Severe n (%)** | **Total n (%)** | **Mild n (%)** | **Moderate n (%)** | **Severe n (%)** | **Total n (%)** | **Mild n (%)** | **Moderate n (%)** | **Severe n (%)** | **Total n (%)** |
| **Patients with at least one TEAE** | 11 (23.40) | 3 (6.38) | 3 (6.38) | 17 (36.17) | 2 (22.22) | 1 (11.11) | 1 (11.11) | 4 (44.44) | 13 (23.21) | 4 (7.14) | 4 (7.14) | 21 (37.50) |
| *System Organ Class /*  Preferred Term |  |  |  |  |  |  |  |  |  |  |  |  |
| *Cardiac disorders* | 0 | 0 | 0 | 0 | 0 | 0 | 1 (11.11) | 1 (11.11) | 0 | 0 | 1 (1.79) | 1 (1.79) |
| Cardiac arrest | 0 | 0 | 0 | 0 | 0 | 0 | 1 (11.11) | 1 (11.11) | 0 | 0 | 1 (1.79) | 1 (1.79) |
| *Ear and labyrinth disorders* | 1 (2.13) | 0 | 0 | 1 (2.13) | 0 | 0 | 0 | 0 | 1 (1.79) | 0 | 0 | 1 (1.79) |
| Otolithiasis | 1 (2.13) | 0 | 0 | 1 (2.13) | 0 | 0 | 0 | 0 | 1 (1.79) | 0 | 0 | 1 (1.79) |
| *Gastrointestinal disorders* | 1 (2.13) | 0 | 0 | 1 (2.13) | 2 (22.22) | 0 | 0 | 2 (22.22) | 3 (5.36) | 0 | 0 | 3 (5.36) |
| Abdominal pain | 0 | 0 | 0 | 0 | 1 (11.11) | 0 | 0 | 1 (11.11) | 1 (1.79) | 0 | 0 | 1 (1.79) |
| Diarrhoea | 1 (2.13) | 0 | 0 | 1 (2.13) | 0 | 0 | 0 | 0 | 1 (1.79) | 0 | 0 | 1 (1.79) |
| Vomiting | 0 | 0 | 0 | 0 | 1 (11.11) | 0 | 0 | 1 (11.11) | 1 (1.79) | 0 | 0 | 1 (1.79) |
| *General disorders and administration site conditions* | 4 (8.51) | 0 | 1 (2.13) | 5 (10.64) | 0 | 0 | 0 | 0 | 4 (7.14) | 0 | 1 (1.79) | 5 (8.93) |
| Death | 0 | 0 | 1 (2.13) | 1 (2.13) | 0 | 0 | 0 | 0 | 0 | 0 | 1 (1.79) | 1 (1.79) |
| Pyrexia | 4 (8.51) | 0 | 0 | 4 (8.51) | 0 | 0 | 0 | 0 | 4 (7.14) | 0 | 0 | 4 (7.14) |
| *Hepatobiliary disorders* | 0 | 0 | 0 | 0 | 0 | 1 (11.11) | 0 | 1 (11.11) | 0 | 1 (1.79) | 0 | 1 (1.79) |
| Hyperbilirubinaemia | 0 | 0 | 0 | 0 | 0 | 1 (11.11) | 0 | 1 (11.11) | 0 | 1 (1.79) | 0 | 1 (1.79) |
| *Infections and infestations* | 5 (10.64) | 2 (4.26) | 0 | 7 (14.89) | 0 | 0 | 0 | 0 | 5 (8.93) | 2 (3.57) | 0 | 7 (12.50) |
| COVID-19 | 1 (2.13) | 0 | 0 | 1 (2.13) | 0 | 0 | 0 | 0 | 1 (1.79) | 0 | 0 | 1 (1.79) |
| Influenza | 3 (6.38) | 0 | 0 | 3 (6.38) | 0 | 0 | 0 | 0 | 3 (5.36) | 0 | 0 | 3 (5.36) |
| Periorbital cellulitis | 1 (2.13) | 0 | 0 | 1 (2.13) | 0 | 0 | 0 | 0 | 1 (1.79) | 0 | 0 | 1 (1.79) |
| Pneumonia | 0 | 1 (2.13) | 0 | 1 (2.13) | 0 | 0 | 0 | 0 | 0 | 1 (1.79) | 0 | 1 (1.79) |
| Pseudomonas infection | 0 | 1 (2.13) | 0 | 1 (2.13) | 0 | 0 | 0 | 0 | 0 | 1 (1.79) | 0 | 1 (1.79) |
| Staphylococcal infection | 0 | 1 (2.13) | 0 | 1 (2.13) | 0 | 0 | 0 | 0 | 0 | 1 (1.79) | 0 | 1 (1.79) |
| *Injury, poisoning and procedural complications* | 1 (2.13) | 1 (2.13) | 1 (2.13) | 3 (6.38) | 0 | 0 | 0 | 0 | 1 (1.79) | 1 (1.79) | 1 (1.79) | 3 (5.36) |
| Fall | 1 (2.13) | 0 | 0 | 1 (2.13) | 0 | 0 | 0 | 0 | 1 (1.79) | 0 | 0 | 1 (1.79) |
| Femur fracture | 0 | 0 | 1 (2.13) | 1 (2.13) | 0 | 0 | 0 | 0 | 0 | 0 | 1 (1.79) | 1 (1.79) |
| Head injury | 0 | 1 (2.13) | 0 | 1 (2.13) | 0 | 0 | 0 | 0 | 0 | 1 (1.79) | 0 | 1 (1.79) |
| Infusion related reaction | 1 (2.13) | 0 | 0 | 1 (2.13) | 0 | 0 | 0 | 0 | 1 (1.79) | 0 | 0 | 1 (1.79) |
| *Nervous system disorders* | 0 | 2 (4.26) | 1 (2.13) | 3 (6.38) | 1 (11.11) | 0 | 0 | 1 (11.11) | 1 (1.79) | 2 (3.57) | 1 (1.79) | 4 (7.14) |
| Cerebral haemorrhage | 0 | 0 | 1 (2.13) | 1 (2.13) | 0 | 0 | 0 | 0 | 0 | 0 | 1 (1.79) | 1 (1.79) |
| Presyncope | 0 | 1 (2.13) | 0 | 1 (2.13) | 0 | 0 | 0 | 0 | 0 | 1 (1.79) | 0 | 1 (1.79) |
| Transient ischaemic attack | 0 | 1 (2.13) | 0 | 1 (2.13) | 0 | 0 | 0 | 0 | 0 | 1 (1.79) | 0 | 1 (1.79) |
| Tremor | 0 | 0 | 0 | 0 | 1 (11.11) | 0 | 0 | 1 (11.11) | 1 (1.79) | 0 | 0 | 1 (1.79) |
| *Psychiatric disorders* | 1 (2.13) | 0 | 0 | 1 (2.13) | 1 (11.11) | 0 | 0 | 1 (11.11) | 2 (3.57) | 0 | 0 | 2 (3.57) |
| Disorientation | 0 | 0 | 0 | 0 | 1 (11.11) | 0 | 0 | 1 (11.11) | 1 (1.79) | 0 | 0 | 1 (1.79) |
| Panic attack | 1 (2.13) | 0 | 0 | 1 (2.13) | 0 | 0 | 0 | 0 | 1 (1.79) | 0 | 0 | 1 (1.79) |
| *Reproductive system and breast disorders* | 1 (2.13) | 0 | 0 | 1 (2.13) | 0 | 0 | 0 | 0 | 1 (1.79) | 0 | 0 | 1 (1.79) |
| Genital burning sensation | 1 (2.13) | 0 | 0 | 1 (2.13) | 0 | 0 | 0 | 0 | 1 (1.79) | 0 | 0 | 1 (1.79) |
| *Respiratory, thoracic and mediastinal disorders* | 2 (4.26) | 0 | 0 | 2 (4.26) | 0 | 0 | 0 | 0 | 2 (3.57) | 0 | 0 | 2 (3.57) |
| Cough | 1 (2.13) | 0 | 0 | 1 (2.13) | 0 | 0 | 0 | 0 | 1 (1.79) | 0 | 0 | 1 (1.79) |
| Respiratory failure | 1 (2.13) | 0 | 0 | 1 (2.13) | 0 | 0 | 0 | 0 | 1 (1.79) | 0 | 0 | 1 (1.79) |
| *Vascular disorders* | 1 (2.13) | 1 (2.13) | 0 | 2 (4.26) | 0 | 0 | 0 | 0 | 1 (1.79) | 1 (1.79) | 0 | 2 (3.57) |
| Hypertension | 1 (2.13) | 0 | 0 | 1 (2.13) | 0 | 0 | 0 | 0 | 1 (1.79) | 0 | 0 | 1 (1.79) |
| Venous thrombosis | 0 | 1 (2.13) | 0 | 1 (2.13) | 0 | 0 | 0 | 0 | 0 | 1 (1.79) | 0 | 1 (1.79) |
| *TEAE = Treatment Emergent Adverse Event.*  *Percentages were computed on patients belonging to the Enrolled population within each considered group. Cohort A is consisting of Pompe disease patients receiving Myozyme in a home-care setting, while Cohort B is composed of MPS-I patients receiving Aldurazyme in a home-care setting. Patients with more than one TEAE or multiple occurrences of the same TEAE were counted only once in the System Organ Class, Preferred Term category or maximum severity grade.* | | | | | | | | | | | | |

S. Table 3: Results of Physical Examination in Enrolled Population

| **Screening**  **EoS** | **Cohort A (N=47)** | | | | **Cohort B (N=9)** | | | | **Total (N=56)** | | | |
| --- | --- | --- | --- | --- | --- | --- | --- | --- | --- | --- | --- | --- |
|  | **Normal** | **Abnormal NCS** | **Abnormal CS** | **Total ^a^** | **Normal** | **Abnormal NCS** | **Abnormal CS** | **Total ^a^** | **Normal** | **Abnormal NCS** | **Abnormal CS** | **Total ^a^** |
| **General appearance** | | | | | | | | | | | | |
| Normal | 27 (96.43) | 1 (100.00) | 2 (100.00) | 30 (96.77) | 4 (100.00) | 0 | 0 | 4 (80.00) | 31 (96.88) | 1 (100.00) | 2 (66.67) | 34 (94.44) |
| Abnormal NCS | 0 | 0 | 0 | 0 | 0 | 0 | 0 | 0 | 0 | 0 | 0 | 0 |
| Abnormal CS | 1 (3.57) | 0 | 0 | 1 (3.23) | 0 | 0 | 1 (100.00) | 1 (20.00) | 1 (3.13) | 0 | 1 (33.33) | 2 (5.56) |
| Total ^a^ | 28 (90.32) | 1 (3.23) | 2 (6.45) | 31 (65.96) | 4 (80.00) | 0 | 1 (20.00) | 5 (55.56) | 32 (88.89) | 1 (2.78) | 3 (8.33) | 36 (64.29) |
| **Eyes, ears, nose and throat** | | | | | | | | | | | | |
| Normal | 15 (100.00) | 0 | 0 | 15 (71.43) | 1 (50.00) | 1 (100.00) | 0 | 2 (66.67) | 16 (94.12) | 1 (16.67) | 0 | 17 (70.83) |
| Abnormal NCS | 0 | 4 (80.00) | 0 | 4 (19.05) | 0 | 0 | 0 | 0 | 0 | 4 (66.67) | 0 | 4 (16.67) |
| Abnormal CS | 0 | 1 (20.00) | 1 (100.00) | 2 (9.52) | 1 (50.00) | 0 | 0 | 1 (33.33) | 1 (5.88) | 1 (16.67) | 1 (100.00) | 3 (12.50) |
| Total ^a^ | 15 (71.43) | 5 (23.81) | 1 (4.76) | 21 (44.68) | 2 (66.67) | 1 (33.33) | 0 | 3 (33.33) | 17 (70.83) | 6 (25.00) | 1 (4.17) | 24 (42.86) |
| **Cardiovascular** | | | | | | | | | | | | |
| Normal | 19 (95.00) | 0 | 0 | 19 (95.00) | 1 (50.00) | 1 (100.00) | 1 (100.00) | 3 (75.00) | 20 (90.91) | 1 (100.00) | 1 (100.00) | 22 (91.67) |
| Abnormal NCS | 1 (5.00) | 0 | 0 | 1 (5.00) | 1 (50.00) | 0 | 0 | 1 (25.00) | 2 (9.09) | 0 | 0 | 2 (8.33) |
| Abnormal CS | 0 | 0 | 0 | 0 | 0 | 0 | 0 | 0 | 0 | 0 | 0 | 0 |
| Total^a^ | 20 (100.00) | 0 | 0 | 20 (42.55) | 2 (50.00) | 1 (25.00) | 1 (25.00) | 4 (44.44) | 22 (91.67) | 1 (4.17) | 1 (4.17) | 24 (42.86) |
| **Respiratory** |  |  |  |  |  |  |  |  |  |  |  |  |
| Normal | 8 (66.67) | 0 | 0 | 8 (36.36) | 3 (100.00) | 0 | 0 | 3 (100.00) | 11 (73.33) | 0 | 0 | 11 (44.00) |
| Abnormal NCS | 1 (8.33) | 3 (50.00) | 2 (50.00) | 6 (27.27) | 0 | 0 | 0 | 0 | 1 (6.67) | 3 (50.00) | 2 (50.00) | 6 (24.00) |
| Abnormal CS | 3 (25.00) | 3 (50.00) | 2 (50.00) | 8 (36.36) | 0 | 0 | 0 | 0 | 3 (20.00) | 3 (50.00) | 2 (50.00) | 8 (32.00) |
| Total^a^ | 12 (54.55) | 6 (27.27) | 4 (18.18) | 22 (46.81) | 3 (100.00) | 0 | 0 | 3 (33.33) | 15 (60.00) | 6 (24.00) | 4 (16.00) | 25 (44.64) |
| **Abdomen** |  |  |  |  |  |  |  |  |  |  |  |  |
| Normal | 14 (100.00) | 0 | 3 (60.00) | 17 (85.00) | 1 (50.00) | 0 | 1 (50.00) | 2 (50.00) | 15 (93.75) | 0 | 4 (57.14) | 19 (79.17) |
| Abnormal NCS | 0 | 1 (100.00) | 2 (40.00) | 3 (15.00) | 1 (50.00) | 0 | 0 | 1 (25.00) | 1 (6.25) | 1 (100.00) | 2 (28.57) | 4 (16.67) |
| Abnormal CS | 0 | 0 | 0 | 0 | 0 | 0 | 1 (50.00) | 1 (25.00) | 0 | 0 | 1 (14.29) | 1 (4.17) |
| Total^a^ | 14 (70.00) | 1 (5.00) | 5 (25.00) | 20 (42.55) | 2 (50.00) | 0 | 2 (50.00) | 4 (44.44) | 16 (66.67) | 1 (4.17) | 7 (29.17) | 24 (42.86) |
| **Urogenital** |  |  |  |  |  |  |  |  |  |  |  |  |
| Normal | 12 (100.00) | 0 | 1 (50.00) | 13 (92.86) | 0 | 0 | 0 | 0 | 12 (100.00) | 0 | 1 (50.00) | 13 (92.86) |
| Abnormal NCS | 0 | 0 | 0 | 0 | 0 | 0 | 0 | 0 | 0 | 0 | 0 | 0 |
| Abnormal CS | 0 | 0 | 1 (50.00) | 1 (7.14) | 0 | 0 | 0 | 0 | 0 | 0 | 1 (50.00) | 1 (7.14) |
| Total^a^ | 12 (85.71) | 0 | 2 (14.29) | 14 (29.79) | 0 | 0 | 0 |  | 12 (85.71) | 0 | 2 (14.29) | 14 (25.00) |
| **Neurological** |  |  |  |  |  |  |  |  |  |  |  |  |
| Normal | 10 (52.63) | 2 (25.00) | 1 (14.29) | 13 (38.24) | 2 (100.00) | 0 | 0 | 2 (100.00) | 12 (57.14) | 2 (25.00) | 1 (14.29) | 15 (41.67) |
| Abnormal NCS | 4 (21.05) | 6 (75.00) | 2 (28.57) | 12 (35.29) | 0 | 0 | 0 | 0 | 4 (19.05) | 6 (75.00) | 2 (28.57) | 12 (33.33) |
| Abnormal CS | 5 (26.32) | 0 | 4 (57.14) | 9 (26.47) | 0 | 0 | 0 | 0 | 5 (23.81) | 0 | 4 (57.14) | 9 (25.00) |
| Total^a^ | 19 (55.88) | 8 (23.53) | 7 (20.59) | 34 (72.34) | 2 (100.00) | 0 | 0 | 2 (22.22) | 21 (58.33) | 8 (22.22) | 7 (19.44) | 36 (64.29) |
| **Musculoskeletal** |  |  |  |  |  |  |  |  |  |  |  |  |
| Normal | 3 (42.86) | 0 | 0 | 3 (8.33) | 1 (100.00) | 0 | 0 | 1 (25.00) | 4 (50.00) | 0 | 0 | 4 (10.00) |
| Abnormal NCS | 3 (42.86) | 6 (54.55) | 8 (44.44) | 17 (47.22) | 0 | 2 (66.67) | 0 | 2 (50.00) | 3 (37.50) | 8 (57.14) | 8 (44.44) | 19 (47.50) |
| Abnormal CS | 1 (14.29) | 5 (45.45) | 10 (55.56) | 16 (44.44) | 0 | 1 (33.33) | 0 | 1 (25.00) | 1 (12.50) | 6 (42.86) | 10 (55.56) | 17 (42.50) |
| Total^a^ | 7 (19.44) | 11 (30.56) | 18 (50.00) | 36 (76.60) | 1 (25.00) | 3 (75.00) | 0 | 4 (44.44) | 8 (20.00) | 14 (35.00) | 18 (45.00) | 40 (71.43) |
| **Lymph nodes** |  |  |  |  |  |  |  |  |  |  |  |  |
| Normal | 10 (90.91) | 0 | 0 | 10 (90.91) | 1 (100.00) | 0 | 0 | 1 (100.00) | 11 (91.67) | 0 | 0 | 11 (91.67) |
| Abnormal NCS | 1 (9.09) | 0 | 0 | 1 (9.09) | 0 | 0 | 0 | 0 | 1 (8.33) | 0 | 0 | 1 (8.33) |
| Abnormal CS | 0 | 0 | 0 | 0 | 0 | 0 | 0 | 0 | 0 | 0 | 0 | 0 |
| Total^a^ | 11 (100.00) | 0 | 0 | 11 (23.40) | 1 (100.00) | 0 | 0 | 1 (11.11) | 12 (100.00) | 0 | 0 | 12 (21.43) |
| **Dermatology** |  |  |  |  |  |  |  |  |  |  |  |  |
| Normal | 13 (100.00) | 1 (100.00) | 0 | 14 (100.00) | 1 (100.00) | 0 | 0 | 1 (50.00) | 14 (100.00) | 1 (50.00) | 0 | 15 (93.75) |
| Abnormal NCS | 0 | 0 | 0 | 0 | 0 | 1 (100.00) | 0 | 1 (50.00) | 0 | 1 (50.00) | 0 | 1 (6.25) |
| Abnormal CS | 0 | 0 | 0 | 0 | 0 | 0 | 0 | 0 | 0 | 0 | 0 | 0 |
| Total^a^ | 13 (92.86) | 1 (7.14) | 0 | 14 (29.79) | 1 (50.00) | 1 (50.00) | 0 | 2 (22.22) | 14 (87.50) | 2 (12.50) | 0 | 16 (28.57) |
| **Other** |  |  |  |  |  |  |  |  |  |  |  |  |
| Normal | 5 (100.00) | 0 | 0 | 5 (100.00) | 0 | 0 | 0 | 0 | 5 (100.00) | 0 | 0 | 5 (100.00) |
| Abnormal NCS | 0 | 0 | 0 | 0 | 0 | 0 | 0 | 0 | 0 | 0 | 0 | 0 |
| Abnormal CS | 0 | 0 | 0 | 0 | 0 | 0 | 0 | 0 | 0 | 0 | 0 | 0 |
| Total^a^ | 5 (100.00) | 0 | 0 | 5 (10.64) | 0 | 0 | 0 |  | 5 (100.00) | 0 | 0 | 5 (8.93) |

*CS= Clinically significant, EOS:End of Study NCS= Not clinically significant*

*Only patients belonging to the Enrolled population who performed physical examination assessments both at screening and post screening visits were considered.
Percentages were computed on patients belonging to the Enrolled population within each assessment’s result.
a Percentages were computed within patients who underwent physical examination assessments both at screening and post screening visits.*
